# Correction: EDOT-based conjugated polymers accessed *via* C–H direct arylation for efficient photocatalytic hydrogen production

**DOI:** 10.1039/d2sc90026b

**Published:** 2022-02-11

**Authors:** Zhi-Rong Tan, Yu-Qin Xing, Jing-Zhao Cheng, Guang Zhang, Zhao-Qi Shen, Yu-Jie Zhang, Guangfu Liao, Long Chen, Shi-Yong Liu

**Affiliations:** Jiangxi Provincial Key Laboratory of Functional Molecular Materials Chemistry, Department of Chemistry, Jiangxi University of Science and Technology Ganzhou 341000 China chelsy@zju.edu.cn chelsy@jxust.edu.cn; Department of Chemistry, Tianjin Key Laboratory of Molecular Optoelectronic Sciences, Tianjin University Tianjin 300072 China; Engineering Research Center of Nano-Geomaterials of Ministry of Education, China University of Geosciences Wuhan 430074 China

## Abstract

Correction for ‘EDOT-based conjugated polymers accessed *via* C–H direct arylation for efficient photocatalytic hydrogen production’ by Zhi-Rong Tan *et al.*, *Chem. Sci.*, 2022, DOI: 10.1039/d1sc05784g.

The authors regret that incorrect details were cited in the following sections of text from the original article.

In the section heading titled ‘Photocatalytic H_2_ production and mechanism analysis’, for the sentence ‘Fig. 4b shows that the CPs dispersed in AA/H_2_O/NMP exhibit 1.5–44 times higher HER than the dispersions in AA/H_2_O/MeOH, which are mainly ascribed to the exfoliation effect in NMP (Fig. S2, S4 and S5†),^37^ yielding colloidal CPs that possess more exposed active sites and a shorter migration distance of charge carriers compared to their bulk counterparts in MeOH’, the correct version should cite ref. 36 instead of ref. 37. The correct details for this citation are given below as ref. 1.

Incorrect details were also given in the section heading titled ‘C–H direct arylation *vs.* classical Stille coupling’, for the sentence ‘More impressively, the PHP activities of both DArP-derived BSO_2_–EDOT and DBT–EDOT are superior to those of their Stille-derived counterparts, *i.e*., 0.95 *vs.* 0.91 mmol h^−1^ for BSO_2_–EDOT and St–BSO_2_–EDOT, and 0.39 *vs.* 0.26 mmol h^−1^ for DBT–EDOT and St–DBT–EDOT (Fig. S13†), which were contrary to our previous results.^30^,’ the correct version should cite ref. 29 instead of ref. 30. The correct details for this citation are given below as ref. 2.

The Royal Society of Chemistry apologises for these errors and any consequent inconvenience to authors and readers.

## Supplementary Material
